# Electrical Properties Enhancement of Carbon Nanotube Yarns by Cyclic Loading

**DOI:** 10.3390/molecules25204824

**Published:** 2020-10-20

**Authors:** Orli Weizman, Joey Mead, Hanna Dodiuk, Samuel Kenig

**Affiliations:** 1 Department of Plastics Engineering, University of Massachusetts Lowell, Lowell, MA 01854, USA; Orli_weizman@student.uml.edu; 2 Department of Polymer Materials Engineering, Shenkar College of Engineering and Design, Ramat Gan 52526, Israel; hannad@shenkar.ac.il (H.D.); SamKenig@shenkar.ac.il (S.K.)

**Keywords:** carbon nanotube yarns, electrical conductivity, cyclic loading

## Abstract

Carbon nanotube yarns (CNTYs) possess low density, high conductivity, high strength, and moderate flexibility. These intrinsic properties allow them to be a preferred choice for use as conductive elements in high-performance composites. To fully exploit their potential as conductive reinforcing elements, further improvement in their electrical conductivity is needed. This study demonstrates that tensile cyclic loading under ambient conditions improves the electrical conductivity of two types of CNTYs. The results showed that the electrical resistance of untreated CNTYs was reduced by 80% using cyclic loading, reaching the resistance value of the drawn acid-treated CNTYs. Scanning electron microscopy showed that cyclic loading caused orientation and compaction of the CNT bundles that make up the CNTYs, resulting in significantly improved electrical conductivity of the CNTYs. Furthermore, the elastic modulus was increased by 20% while preserving the tensile strength. This approach has the potential to replace the environmentally unfriendly acid treatment currently used to enhance the conductivity of CNTYs.

## 1. Introduction

Carbon nanotube (CNT) yarns (CNTYs) are long, flexible, conductive, and strong materials composed of assembled CNT bundles. They could be used in a broad range of applications due to their lightweight and ability to provide enhanced mechanical and electrical performance. In particular, the use of CNTYs as a conducting component could lead to an advanced generation of electronics, such as flexible displays, electronic paper, smart packages, skin-like sensors, wearable electronics, and medical implants, that could change the role of electronics in our daily lives [1,2,3,4,5]. Nevertheless, some fundamental challenges remain in translating the inherent properties of the CNT building blocks into macro-applications.

CNTYs are produced by various methods, including chemical vapor deposition, where carbon is deposited on specific and active catalysts that control the formation of tubular carbon nanostructures. The resulting forest of CNTs is drawn to produce bundles of CNTs held together by π-π interactions [6,7]. The CNT bundles display relatively low mechanical strength than the individual CNTs, due to the low interconnections and weak bonding between the nanotubes [8]. Several approaches have been used to improve the strength of the CNT bundles such as chemical surface coatings, electron beam irradiation treatment [9,10], and chemical cross-linking [11]. These treatments lead to a decrease in the CNTs’ electrical conductivity [12,13]. Doping is another approach to improve the electrical conductivity of CNT bundles. For instance, Iodine doped CNT bundles display a higher specific conductivity than that of metals [14]. Although these approaches improve the properties of the CNTYs, they require chemical post-treatment. Twisting of CNT bundles to produce yarns has received much attention, mainly because of its simplicity and capability of producing long continuous fibers with improved properties. In this case, the CNT bundles are condensed either by using a solvent or through twisting or both [8,15,16,17]. The twisting enhances the interaction between CNT bundles, leading to a higher degree of packing and inter bundle friction enhancing the capability of the material to withstand tensile stresses [7,16,18,19]. Typical solvents, such as acetone, ethanol, methanol, and dichloroethane, condense the CNTYs [20,21]. Typical post-treatment consists of acid treatment, which is useful in enhancing the electrical conductivity of CNT bundles [22]. To further improve the tensile properties of CNTYs, they are stacked into multi-ply yarns, which are drawn and twisted together [13].

Furthermore, it has been found that post-treatment using stretching of CNTYs in the presence of acids, such as chlorosulfonic acid, triflic acid, and fluorosulfuric acid, can dramatically improve the tensile strength and electrical conductivity of the yarns due to the alignment of the CNT bundles [23,24]. Although this post-treatment can improve the strength and electrical conductivity of the CNTYs, these strong acids present environmental issues. It may affect the composition of the nanotubes.

In this work, an innovative strategy, based on uniaxial cyclic loading of commercially available CNTYs without the use of acids, was investigated. The proposed process has been proposed for a patent application [25]. Using a cyclic loading mechanism with increasing strains leads to the alignment of the CNTs and results in increased electrical conductivities for as received untreated CNTYs and acid-treated CNTYs. Furthermore, the stiffness is increased without compromising strength. This chemical-free mechanical based method resulted in exceptional electrical properties equivalent to that of the acid-treated aligned CNTYs and yielding an environmentally friendly process to enhance the CNTYs properties.

## 2. Experimental

### 2.1. Materials

Nanocomp Technologies, Inc. (Merrimack, New Hampshire) provided CNTYs (Miralon^®^ yarn). The CNTYs are composed of aligned bundles of CNTs, hundreds of microns in diameter, and millimeters in length. The fiber is manufactured by chemical vapor deposition (CVD) [26]. The manufacturer provided two types of CNTYs: a direct-spun untreated single-ply CNTYs with a 10 tex (10 g/km) linear density and a diameter of ~150 microns (Nanocomp A-series), as well as post-treated four-ply CNTYs that were stretched in the presence of acids by the manufacturer with a 29 tex linear density and a diameter of ~250 microns (Nanocomp C-series).

### 2.2. Methods

The yarns’ tensile behavior was studied using a Mechanical Tester (4466 Instron machine), with a 2 kN loading capacity. The displacement of the grips was used to calculate the strain, assuming a gauge length of 70 mm (original distance between grips). The elongation rate used for this test was 10 mm/min. Electrical resistance was measured simultaneously while stretching the yarns at a strain rate of 5 mm/min. For cyclic testing, samples were subjected to 100 cycles of stretching/relaxation to predetermined strain values at 5 mm/min. At least three samples were characterized under the same conditions in this study.

As indicated, electrical resistance was measured simultaneously with stress-strain measurements during the extension-relaxation cycles. The electrical properties of CNTYs were measured using a two-probe electrical resistance device (FLUKE 179 multimeter). The measuring probes were connected by conductive tapes (copper foil) to the yarn to achieve good contact between the CNTYs and the probes. An insulating layer between the CNTYs/conductive tape and the clamps was included for insulation. CNTYs surface morphology analysis was carried out using scanning electron microscopy (JEOL JSM 6390). The applied accelerating voltage was 5 kV.

It is important to mention that there are many factors during the production of CNTYs that can influence the final properties of the yarns such as variations in the CNT yarn diameter, CNT length, presence of catalyst impurities, and nanoscale voids between the CNTs, that could affect the overall response of the CNTYs [24,27]. These factors may lead to variability in the measurements. For each test, at least three samples were tested. The results shown represent typical behavior for each type of yarn. Properties calculated using the measured cross-sectional area (datasheets from Nanocomp), such as the electrical and mechanical properties (modulus, strength, etc.), are referred to as “apparent” properties. The specimens were also weighed to calculate their specific gravity, which was used to calculate their specific mechanical properties.

## 3. Results and Discussion

The CNTYs used in this study were manufactured using chemical vapor deposition (CVD) and were spun into single-ply yarn or four-ply CNTYs [26]. The four-ply CNTYs were post-treated by the manufacturer by applying unidirectional stretching in the presence of acid to enhance their mechanical and electrical properties. 

**Figure 1:**
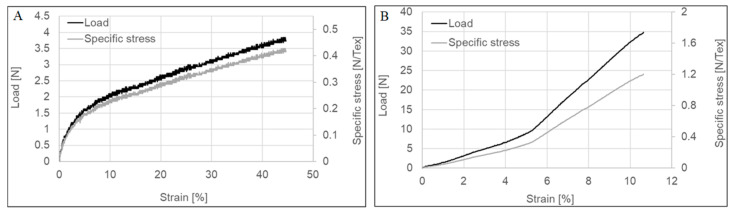
Stress–strain curve of untreated CNTY (**A**) and post-treated CNTY (**B**).

Tensile tests of the untreated and post-treated CNTYs exhibit stress-strain curves, as shown in Figure 1 and summarized in Table 1. The two types of yarns showed different behaviors for a single tensile test. The density normalized strength is in the range of 0.41–0.45 GPa g^−1^ cm^3^ (N/Tex) for untreated yarn, while the density normalized strength of the post-treated CNTYs is in the range of 1.1–1.2 GPa g^−1^ cm^3^ (N/Tex). The maximum tensile strain to failure was significantly lower for the post-treated CNTYs than the untreated ones. The slope of the stress-strain curves of the untreated CNTYs (apparent modulus) is initially high but becomes lower above 5% strain. It is attributed to the entangled and misaligned CNT bundles becoming oriented in the stretch direction, and thus an initially high engineering modulus is observed. Above 5% strain, in the apparent tensile modulus decreases, attributable to slippage and reorientation of the CNT bundles [28].

In comparison, the post-treated CNTYs exhibited a low initial modulus due to the CNT bundles’ reorientation. Above 5% strain, a higher modulus was observed, caused by increased orientation of the bundles [29]. The difference in the stress-strain behavior of the two types of CNTYs indicates that pre-stretching of CNTYs in the presence of acid enhances the inter-CNT interactions and alignment of the CNT bundles, resulting in a lower strain to failure at higher failure stress, coupled with a higher modulus compared to the untreated CNTYs [30]. Phase behavior of CNTs treated by superacids has been well described in the literature. It was found that the superacid treatment of CNTs results in a liquid crystalline phase that can be easily aligned in the draw direction [31,32].

**Figure 2:**
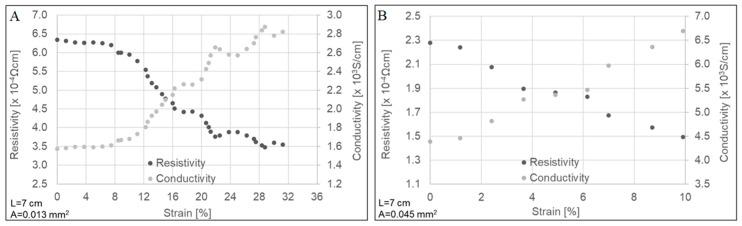
Electrical properties as a function of strain for untreated CNTYs (**A**) and post-treated CNTYs (**B**). L and A represent the yarn’s length between the electrodes and the average cross-sectional area of the yarn, respectively.

Figure 2 displays the electrical resistivity and conductivity measurements during the uniaxial stretching of both types of CNTYs. Higher electrical conductivity was observed for the post-treated CNTYs before stretching compared to the untreated CNTYs (1.5 × 10^3^ vs. 4.4 × 10^3^ S/cm). These results confirm that the post-treatment of the CNTYs by stretching in the presence of an acid promotes an alignment of the CNT bundles, which increases the contact between the nanotubes and reduces their electrical resistance [6,33]. The electrical properties of both yarns were measured under tension up to failure. For both yarns, the electrical conductivity increased with strain and reached a value of 2.8 × 10^3^ S/cm for the untreated CNTYs and a value of 6.7 × 10^3^ S/cm for the post-treated CNTYs. The enhanced electrical conductivity can be rationalized by the alignment of CNTs bundles coupled with compaction of the yarns, leading to better electron transfer between the tubes [30,34,35]. Inspecting the modulus curves for the untreated CNTYs in Figure 1A, the modulus shows significant change above 5% strain, attributed to the alignment of the CNTYs. The electrical properties shown in Figure 2A show a small change in conductivity below 5% strain, but above 5% strain the conductivity increases. This suggests a strain level of 5% is needed to produce the necessary rearrangement of the carbon nanotubes for improved conductivity in the case of the untreated CNTYs. A similar phenomenon was observed for the post-treated CNTYs at 1% strain (Figure 2B).

**Figure 3:**
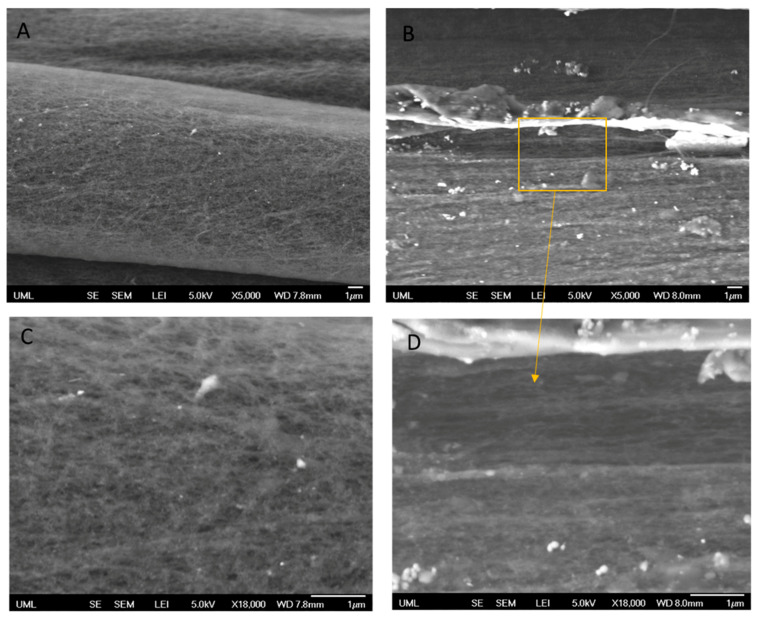
Untreated CNTY (side view) after uniaxial stretching to 2% strain (**A** + **C**) and 30% strain (**B** + **D**). The orange square focuses on a specific area of the image.

**Figure 4:**
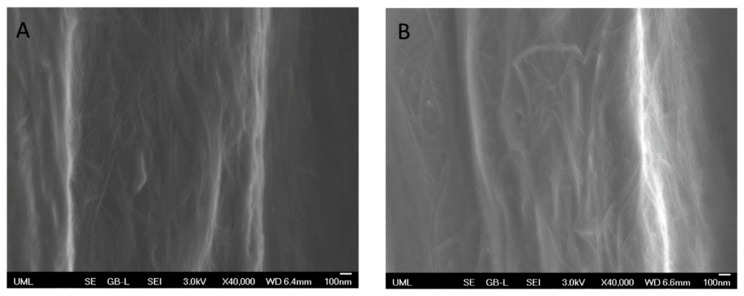
Post treated CNTY (side view) before stretching (**A**) and after uniaxial stretching to 5% strain (**B**).

Figure 3 illustrates the SEM images of untreated CNTYs after stretching to 2% strain and 30% strain. The SEM images clearly indicate the difference in the morphology between both samples induced by stretching. The structure of the untreated CNTYs was significantly changed with increasing the strain from 2% to 30%, displaying an oriented structure of the CNTs under the higher strain. The higher alignment and compaction of the CNTs bundles result in enhancement of the yarns’ tensile modulus and electrical properties. In contrast, the post-treated CNTYs SEM images did not show a clear difference in morphology following stretching (Figure 4A,B). 

### Cyclic Loading of CNTYs

**Figure 5:**
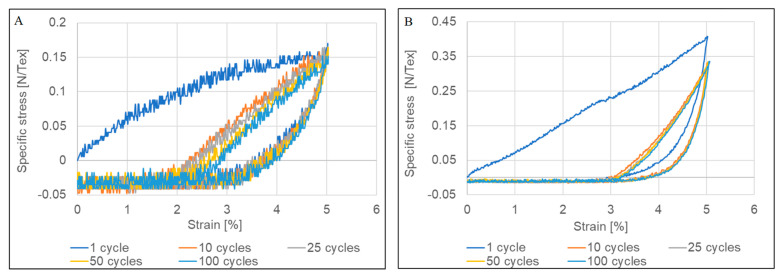
Tensile properties of (**A**) untreated CNTYs and (**B**) post-treated CNTYs under cyclic loading; 100 stretching/relaxing cycles were conducted to a constant 5% strain.

The enhanced electrical, as well as the mechanical properties that were demonstrated following a single loading, prompted the study into cyclic loading with the notion that multiple loadings could lead to further enhancement in properties. Consequently, cyclic tests in tension between 0 and 5% strain for 100 cycles were performed and are presented in Figure 5. As can be observed, the materials showed permanent deformation and a lack of recovery to the original state. Furthermore, hysteresis was apparent due to energy dissipative mechanisms emanating from the energy dissipated in alignment and friction loading CNTYs, similar to other stress softening behavior seen in a variety of other materials [36,37,38]. The accumulated residual deformation reached about 2.5% after 100 cycles for untreated CNTYs and 3% for the post-treated CNTYs. Similar behavior has been previously reported [39,40].

**Figure 6:**
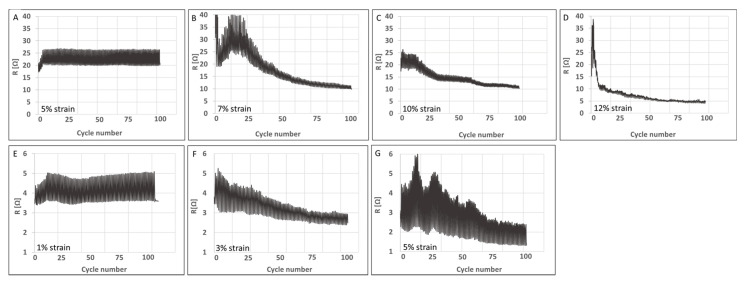
Electrical properties of untreated CNTYs (**A**–**D**) and post-treated CNTYs (**E**–**G**) during cyclic stretching to a different loading strain (100 cycles).

**Figure 7:**
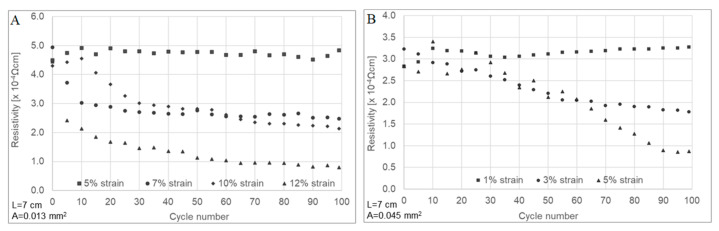
Apparent electrical resistivity of untreated CNTYs (**A**) and post-treated CNTYs (**B**) during cyclic stretching to a different loading strain (100 cycles).

Following this mechanical response, both yarns’ electrical resistance was acquired simultaneously with cyclic loading up to 100 cycles. In the case of untreated CNTYs, the loading was carried out for 5, 7, 10, and 12% cyclic strain and for post-treated CNTYs at 1 to 5% strain. The results are presented in Figure 6. The untreated CNTYs cycled at 5% strain resulted in reversible resistance for loading and unloading over 100 cycles. When the strain was increased to 7%, 10%, and 12%, a unique phenomenon was observed, which has not been previously reported (Figure 6A–D). The electrical resistance of the untreated CNTYs was significantly decreased (up to 80% at 12% strain) with increasing numbers of cycling, ultimately reaching a plateau (Figure 6E–G). The higher the strain, the sooner the plateau was reached. This is consistent with the orientation of the CNTs in the stretch direction and the formation of a denser structure leading to improved electrical properties. The post-treated CNTYs demonstrated a similar trend; however, the resistance change was lower as the post-treated CNTYs were already partially aligned. This simple physical technique of cyclic stretching used to orientate the CNT bundles of the untreated CNTYs resulted in exceptional electrical conductivities similar to those obtained following acid treatment of CNTYs, but without the need for harsh acids [22,24]. It should be mentioned that increasing the cyclic strain levels above 12% for the untreated CNTYs and above 5% for the post-treated CNTYs did not result in additional enhancements in the electrical conductivity. Figure 7 demonstrates the apparent resistivity at the end of each tenth cycle for the yarns presented in Figure 6. It is evident that the electrical resistivity of the untreated CNTYs was significantly decreased due to the cyclic loading (strain >5%), reaching the electrical resistivity value of the post-treated CNTYs (cyclic loading of 5% strain). 

**Figure 8:**
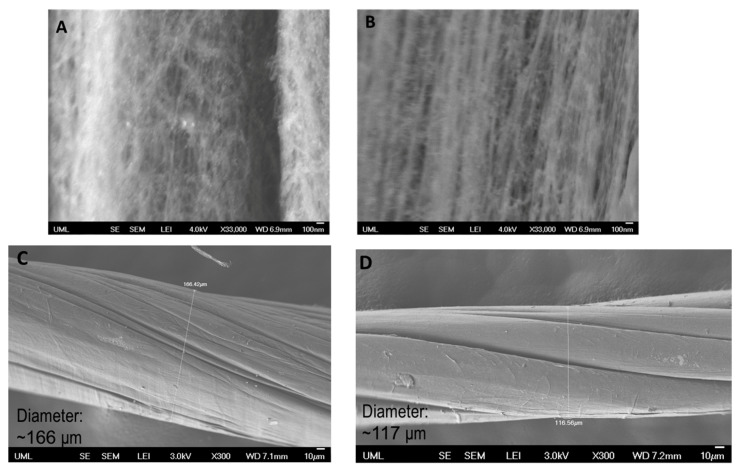
Untreated CNTYs (stretching direction) after 100 cycles to a 5% strain (**A**) and a 12% strain (**B**). Untreated CNTYs diameter (perpendicular to the stretching direction) before (**C**) and after cyclic tension to 12% strain (**D**).

SEM images characterized the morphology of the CNTYs after cyclic loading. The results are presented in Figure 8. In correlation with the electrical resistance results, the surface morphology of the untreated CNTYs changed following cyclic tension above 5% strain. As can be seen in the case of 12% strain cycling, the CNT bundles were oriented in the stretch direction (Figure 8B), assuming an aligned structure. Moreover, the untreated CNTYs diameter was decreased from ~166 μm to ~117 μm following cyclic tension to 12% strain (Figure 8C,D), forming a denser structure. The change of the morphology is consistent with the enhancement in the electrical resistance decrease. In contrast, the post-treated CNTYs did not show a clear difference in morphology following 100 cycles. Their diameter, however, decreased after cyclic tension at 5% strain (~205 μm to ~184 μm) (not shown). 

The mechanical characteristics of the CNTYs after 100 cycles were investigated and compared to the yarns’ mechanical properties following the first cycle of stretching. The results are presented in Table 2. The apparent Young’s modulus of both yarns was increased after the cyclic loading for all stretching levels compared to the first cycle. A more significant increase was observed above 5% strain and above 1% strain for the untreated and treated CNTYs, respectively. These results are in good agreement with the yarns’ enhanced electrical properties, owing to the alignment of the CNTs bundles. Inspecting the specific tensile stress results at the max strain (column 1) for both yarns, while considering the standard deviations obtained, the observed differences in stress at the first cycle versus the 100th cycle at all cyclic strain values do not appear significant. 

**Figure 9:**
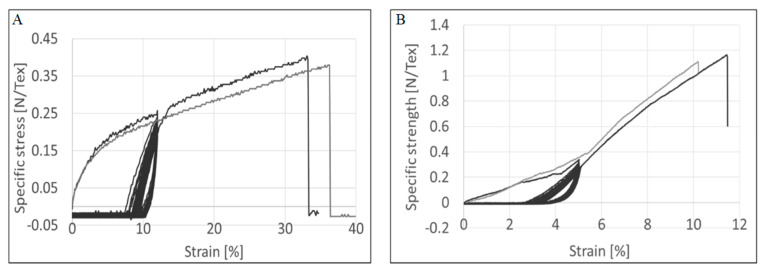
Failure properties of CNTY under uniaxial stretching (gray curve) vs. after 100 cycles of loading (black curve) for untreated CNTY (**A**) and post-treated CNTY (**B**).

Finally, the failure characteristics of the CNTYs after cyclic loading were evaluated by uniaxially loading the samples to failure. Figure 9 compares the failure properties of the treated and the post-treated CNTYs under uniaxial stretching (gray curve) vs. after 100 cycles of loading (black curve showing the cyclic behavior). The test was performed at the highest strain value where the most significant improvement in the electrical properties was observed. As evident from Figure 9, the CNTYs were not significantly affected by the cyclic loading indicating that the yarns’ failure mechanism was not changed as a result of cyclic loading.

## 4. Conclusions

A facile and environmentally friendly method to enhance the electrical property of CNTYs was studied in this work. Tensile cyclic loading demonstrated that the permanent alignment of the CNT bundles comprising the CNTYs could be achieved, leading to a significant increase in the CNTYs electrical conductivity reaching the conductivity levels of the higher-cost and environmentally unfriendly super acid-treated CNTYs. An increased Young’s modulus accompanied the enhanced electrical properties. Morphology investigations using high-resolution SEM indicated that the alignment and compaction of the CNT bundles of the untreated CNTYs after the cyclic loading was the leading cause of their improved electrical performance.

## Figures and Tables

**Table 1:** Mechanical properties of carbon nanotube yarns (CNTYs) during single axial stretching.

	Extension	Load	Specific Stress	Tensile Strain
	(mm)	(N)	(N/Tex)	(%)
Untreated CNTYs	27.4 ± 0.46	3.8 ± 0.04	0.4 ± 0.01	45.7 ± 0.77
Post treated CNTYs	6.4 ± 0.17	33.8 ± 1.34	1.2 ± 0.05	10.6 ± 0.28

**Table 2:** Effects of cyclic loading on the mechanical properties of CNTYs.

	Maximum Strain for Cyclic Stretching [%]	Apparent Young’s Modulus at the First Cycle [N/Tex]	Apparent Young’s Modulus at the 100th Cycle [N/Tex]	Specific STRESS at the first Cycle for the Max. Strain * [N/Tex]	Specific Stress at the 100th Cycle for the Max. Strain * [N/Tex]
Untreated CNTYs	5	7.38 ± 0.38	8.05 ± 0.58	0.17 ± 0.00	0.15 ± 0.01
	7		8.82 ± 0.86	0.20 ± 0.02	0.22 ± 0.04
	10		8.52 ± 0.22	0.23 ± 0.02	0.21 ± 0.08
	12		8.60 ± 0.05	0.24 ± 0.01	0.21 ± 0.04
Post treated CNTYs	1	13.54 ± 1.22	14.63 ± 1.01	0.10 ± 0.08	0.14 ± 0.01
	3		15.60 ± 0.22	0.27 ± 0.10	0.27 ± 0.10
	5		16.86 ± 0.06	0.41 ± 0.14	0.36 ± 0.15

* The specific stress values were the maximum stress values at the specific maximum strain of the cyclic strain (as listed in the first column—5, 7, 10, etc.).
